# Determination of Malignancy Risk Factors Using Gallstone Data and Comparing Machine Learning Methods to Predict Malignancy

**DOI:** 10.3390/jcm14176091

**Published:** 2025-08-28

**Authors:** Sirin Cetin, Ayse Ulgen, Ozge Pasin, Hakan Sıvgın, Meryem Cetin

**Affiliations:** 1 Department of Biostatistics, Faculty of Medicine, Amasya University, Amasya 05100, Türkiye; 2 Department of Mathematics and Physics, School of Science and Technology, Nottingham Trent University, Nottingham N11 8NS, UK; 3 Department of Biostatistics, Faculty of Medicine, Girne American University, Karmi 99428, Cyprus; 4 Department of Biostatistics, Hamidiye Medical Faculty Health Science University, İstanbul 34093, Türkiye; 5 Department of Internal Medicine, Faculty of Medicine, Tokat Gaziosmanpasa University, Tokat 60250, Türkiye; 6 Department of Microbiology, Faculty of Medicine, Amasya University, Amasya 05100, Türkiye

**Keywords:** gallstone disease, malignancy, machine learning, predictive modeling, clinical insights

## Abstract

**Background/Objectives:** Gallstone disease, a prevalent and costly digestive system disorder, is influenced by multifactorial risk factors, some of which may predispose to malignancy. This study aims to evaluate the association between gallstone disease and malignancy using advanced machine learning (ML) algorithms. **Methods:** A dataset comprising approximately 1000 patients was analyzed, employing six ML methods: random forests (RFs), support vector machines (SVMs), multi-layer perceptron (MLP), MLP with PyTorch 2.3.1 (MLP_PT), naive Bayes (NB), and Tabular Prior-data Fitted Network (TabPFN). Comparative performance was assessed using Pearson correlation, sensitivity, specificity, Kappa, receiver operating characteristic (ROC), area under curve (AUC), and accuracy metrics. **Results:** Our results revealed that age, body mass index (BMI), and history of HRT were the most significant predictors of malignancy. Among the ML models, TabPFN emerged as the most effective, achieving superior performance across multiple evaluation criteria. **Conclusions:** This study highlights the potential of leveraging cutting-edge ML methodologies to uncover complex relationships in clinical datasets, offering a novel perspective on gallstone-related malignancy. By identifying critical risk factors and demonstrating the efficacy of TabPFN, this research provides actionable insights for predictive modeling and personalized patient management in clinical practice.

## 1. Introduction

Gallstone disease is one of the most common and costly digestive system diseases. The frequency of the disease varies according to population and gender. Studies have shown that the risk of gallstone formation is three times higher in women than in men. In Western populations, the incidence of gallbladder disease in the adult population is 10–15%. Gallstones are mainly composed of two different types: cholesterol stones and pigment stones. While they can be asymptomatic, those who are symptomatic may present with dyspeptic complaints, acute and chronic cholecystitis, or serious complications. The preferred surgical intervention for gallbladder disease is laparoscopic or open cholecystectomy.

The formation of gallstone disease has been known to involve many risk factors. Studies have shown that the formation of gallstones is associated with race, genetic factors, female gender, age, obesity, rapid weight loss, physical inactivity, alcohol, smoking, rapid weight gain, use of drugs (oral contraceptives, hormone replacement therapies, thiazide diuretics, octreotide, ceftriaxone, etc.), ileal diseases, hemolytic anemias, diabetes mellitus (DM), and hyperlipidemia.

As a general surgeon, managing gallstone disease is a frequent aspect of surgical practice. Understanding the multifactorial etiology of this condition is crucial for effective prevention and treatment. Women, particularly those who are pregnant or on HRT, represent a high-risk group due to hormonal influences on bile composition. These factors not only increase the lithogenicity of bile but also exacerbate gallstone-related symptoms during these periods. Additionally, certain medications like ceftriaxone, which alters bile salt transport, are frequently encountered in clinical scenarios and contribute to gallstone formation.

Recent data also indicates a rising prevalence of gallstone disease in younger age groups, likely driven by increasing rates of obesity and sedentary lifestyles, which are known to be significant contributors to gallstone formation. Furthermore, metabolic syndrome and its associated components, such as insulin resistance and hypertriglyceridemia, have been strongly linked to the development of cholesterol gallstones [1].

The effect of having any form of malignancy on the formation of gallstones has not been clearly established in the literature. In particular, it is thought that weight loss and physical inactivity may be a risk factor for gallstones in individuals with malignancy. Additionally, patients with advanced malignancies often experience altered bile secretion dynamics, secondary to prolonged fasting, parenteral nutrition, or cachexia, further increasing their susceptibility to gallstone formation [1]. Patients undergoing chemotherapy or radiation therapy may experience biliary stasis due to decreased mobility or altered metabolic states, potentially increasing the risk of gallstone disease. Given the interplay between malignancy and gallstone formation, a deeper understanding of these mechanisms is essential for optimizing patient outcomes.

In this study, we aimed to investigate whether the existence of gallstones had any impact on the formation of malignancy, along with the existing risk factors. Six different machine learning methods were used for these analyses: random forests (RFs), support vector machines (SVMs), multi-layer perceptron (MLP), multi-layer perceptron (MLP_PT, i.e., using the PyTorch library (Version 2.3.1)), naive Bayes (NB), and TabPFN. This study aimed to investigate whether gallstone disease contributes to the development of malignancy by leveraging advanced machine learning methods on a clinical dataset. While gallstones are the most common risk factor for gallbladder cancer, the association between gallstones and other cancers is poorly understood. By comparing six different algorithms (including the novel transformer-based TabPFN) and analyzing numerous clinical risk factors (age, BMI, HRT use, etc.), our work fills this gap and offers new insights. The originality of this study lies in applying state-of-the-art ML models to gallstone-related cancer prediction and identifying the most influential predictors, thus providing actionable contributions for future research and patient care.

## 2. Material and Methods

The study has been approved by the Clinical Research Ethics Committee. Retrospective records of patients followed up with the diagnosis of cholelithiasis were examined at the Internal Medicine Clinic of Tokat Gaziosmanpaşa University Faculty of Medicine Hospital. Approximately 1000 patients over 18 years of age with the diagnosis of cholelithiasis were included in the study between 1 January 2011 and 1 December 2020 in Tokat Gazismanpasa University Hospital. Risk factors that could lead to gallstone formation in the patients included in the study were age, gender, obesity, dyslipidemia, diabetes mellitus, cirrhosis of the liver, rapid weight loss, ileal resection or bypass, sedentary lifestyle, hypothyroidism (hashimoto disease), hyperthyroidism (Graves’ disease), gallbladder and other cancers, chronic Hepatitis C virus (HCV), pregnancy, and exogenous estrogens containing oral contraceptives or hormone replacement therapies, ceftriaxone, and statin drugs were determined using the hospital database and the Ministry of Health e-Nabız system (this is an application in Turkey that citizens and health professionals access to view health data collected from health institutions via internet and mobile devices).

### 2.1. Inclusion Criteria for Participation

Between 2011 and 2020, patients aged 18 and above who had been diagnosed with cholelithiasis were recruited for a study at Tokat Gaziosmanpasa University Hospital.

### 2.2. Exclusion Criteria

The exclusion criteria were defined as patients under 18 years of age, patients without sufficient clinical information, patients with incomplete imaging and laboratory data, patients without specified body weight and height in the e-Nabız and hospital records, patients with Anti-HCV levels not measured, and patients with no thyroid function tests performed.

### 2.3. Preprocessing of Data

**Figure 2:**
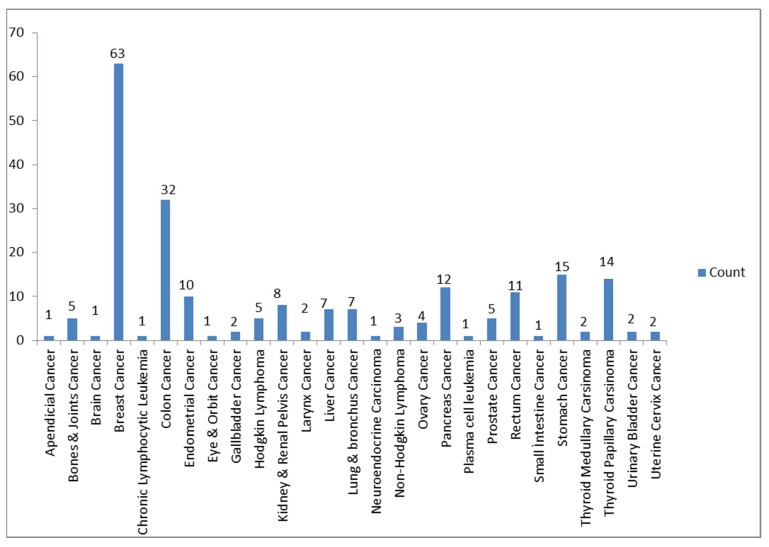
Types of cancer in patients and their distribution.

There are 999 samples in the original data which were grouped into two classes. Each sample contains 14 features, and one label, which is malignity. There are no missing values in the data. One class (i.e., there is malignity) contains 218 samples (Figure 2) and the other class (i.e., when the subject is healthy) contains 781 samples. We made a random split by separating the weights of the data into two classes, named as the training and test data. The test data consisted of 100 elements of which 22 rows are labeled as 1 (there is malignity) where the rest are 0 (the subject is healthy). The training data consisted of 899 rows of which 196 rows are labeled as 1. After we separated the training data, we performed a random oversampling method to avoid bias in the training process. After the oversampling, our training data consisted of 1406 rows, distributed equally in ones and zeros. The entire dataset was randomly split into two subsets, with 80% used for training and 20% for testing, while preserving the class distribution.

### 2.4. Feature Selection

**Figure 3:**
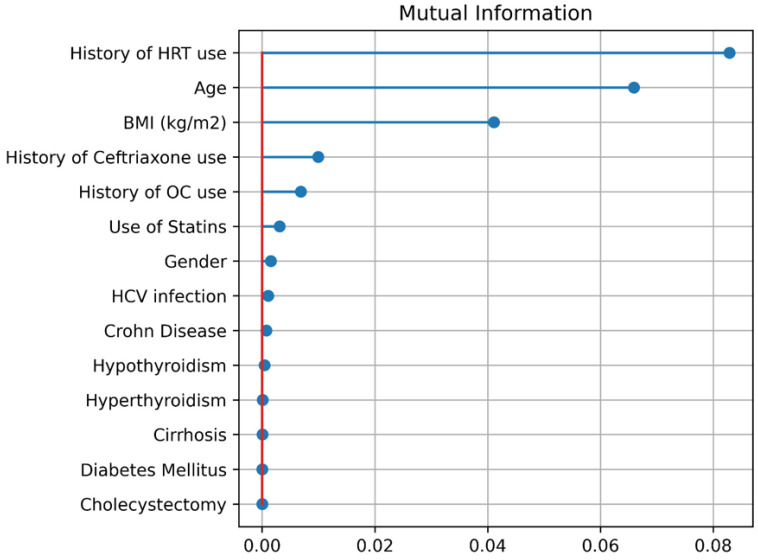
Mutual Information.

We calculated the mutual information for the data set to determine which features were the most relevant ones. Since the data is not very big, we did not perform feature reduction and included every feature in the training processes of machine learning algorithms. It is seen (Figure 3) that history of HRT use is the most important feature followed by age and (BMI).

### 2.5. Machine Learning Algorithms

In this study we considered the following six machine learning (ML) algorithms: random forests (RFs), support vector machines (SVMs), multi-layer perceptron (MLP), multi-layer perceptron (MLP_PT, i.e., using the PyTorch library), naive Bayes (NB), and TabPFN. TabPFN is a recent transformer model that was introduced in July 2022 [2]. For these six methods we trained models to obtain the maximum score on Pearson correlation coefficient, sensitivity, specificity, Kappa, ROC, AUC, and accuracy.

Firstly, RF is a model where several decision trees are created with different configurations. In order to find out what a RF model predicts for a label of a data instance, outputs which are inferred by decision trees are averaged.

Moreover, SVM is another approach to classify data instances. It fundamentally attempts to put a boundary line in a feature space to separate data instances belonging to different classes.

Furthermore, MLP and its variants have become dominant machine learning architecture in recent decades. It takes high-dimensional features as input, and applies transformation processes to infer results for the specified task, such as classification, regression, and so on.

Finally, there is another approach utilized in this study, called TabPFN. It is a recent transformer-based model pre-trained on tabular data, and it was been introduced in July 2022 [2]. A transformer model is a variant of the MLP model that has a self-attention mechanism.

There are some processes applied before training some of the aforementioned models. First, for the RF, values of the column age are binned within different ranges. Moreover, the column age is normalized by standard scaling and the remaining columns are encoded with one-hot encoder before training MLP and SVM models. In order to handle the age column for the NB, two naive Bayes approaches are utilized: Gaussian and Categorical. Gaussian NB only uses the age column while the latter uses the remaining columns. Then, their results are combined together by multiplication. Lastly, there is no preprocess applied to data for TabPFN.

**Figure 1:**
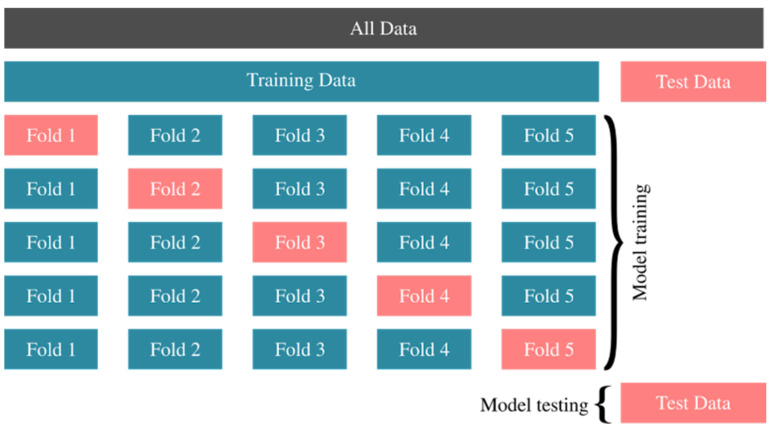
Illustration of the cross-validation process.

We performed 10-fold cross-validation. In Figure 1, we have shown an illustration of 5-fold cross-validation where the oversampling stage is not shown. Oversampling can be considered as a map from training data to oversampled data on which folds are divided. All data is divided into training and test data in the beginning. Then, training data is divided randomly into k subsets; in this case k = 5. In the first row, a ML model is trained on the sum of Fold 2, Fold 3, Fold 4 and Fold 5. After the training takes place, it makes a prediction for the data in Fold 1 and a score is calculated according to its success. The overall success of the model is then averaged for other steps in the cross-validation process. When an algorithm is trained to achieve the best score, it is then applied to the Test Data that we separated at the beginning, and its success is determined according to its ability to correctly predict the Test Data that it has not seen during the training process.

In our study we made use of Python 3.12.3 libraries such as the Scikit framework [3] to train the data, and Optuna [4] to perform hyper-parameter optimization. In handling the data, we used the pandas [5] Python library. In the MLP_PT model, we used PyTorch [6]. Since the training process of ML algorithms depends on random numbers, we set the seed of random number generators to some fixed values in order to obtain reproducible results.

## 3. Results

The mean age of the patients included in the study was 61 ± 14. Relevant descriptive statistics values according to malignancy, gender, BMI, and disease status are summarized in Table 1.

In Table 2, performances of each ML model on different metrics are shown. Note that the ML models are trained separately to maximize each score, that is, for example, the random forest model that maximizes the sensitivity and the random forest model that maximizes specificity do not necessarily share the same hyperparameters. It is seen that TabPFN is the most successful method for five scores and in the sixth one (sensitivity score) it is only just behind the naive Bayes method.

As it is seen in Table 2, the TabPFN algorithm is the most successful. It has the best score for metrics specificity, accuracy, ROC AUC, Kappa score and Pearson score. It is just slightly unsuccessful for the sensitivity score, where the naive Bayes algorithm is the most successful one.

The RF algorithm is the second-best ML algorithm on four out of six metrics. The hyperparameters for each algorithm that have been trained separately to maximize each score can be found in Table 3. In Table 3, higher smoothing values are determined for accuracy, Kappa, and Pearson metrics. In Table 3, Kappa and Pearson metrics require a remarkably lower number of ensemble configurations.

We see a few features that have a higher impact on malignity, according to Figure 3. The top three factors are (1) history of HRT use, (2) age, and (3) BMI. In terms of age, being 55 years or more older as a risk factor.

The six machine-learning models showed diverse predictive performance on malignancy risk (Table 2). Notably, the TabPFN algorithm achieved the highest overall accuracy and discrimination: sensitivity = 0.691, specificity = 0.847, accuracy = 0.814, and AUC = 0.768. In contrast, the naive Bayes model had the highest sensitivity (0.747) but lower specificity (0.713) and accuracy (0.720). Random forest was the second-best performer on four of six metrics (e.g., specificity 0.826, accuracy 0.782), while SVM and standard MLP generally underperformed. DeLong’s statistical test confirmed that TabPFN’s superior AUC (0.768) was significantly higher than that of the next-best model (*p* < 0.05). These findings indicate that the TabPFN model provides a statistically and clinically meaningful improvement in discriminating patients with versus without malignancy. From a clinical standpoint, a model with 77% AUC and high specificity suggests it could reliably rule in malignancy among gallstone patients, whereas the higher-sensitivity NB model might be preferred when minimizing missed cancer cases (at the expense of more false positives). In practice, threshold tuning could tailor the model to specific clinical priorities (screening vs. confirmation). Mutual-information analysis (Figure 3) identified history of HRT use, older age, and higher (BMI) as the most important predictors of malignancy. This pattern aligns with known epidemiology, whereby long-term estrogen (such as in HRT) substantially increases. From Figure 3, it was concluded that age >55 is a risk factor.

Women on HRT often have lithogenic bile, and obesity promotes cholesterol gallstone formation, both of which may predispose to malignancy. Clinically, these results suggest that postmenopausal women on HRT and obese older patients with gallstones warrant especially close surveillance. Overall, the TabPFN model’s robust performance on multiple metrics (high accuracy and AUC) and identification of well-recognized risk factors indicate it could serve as an effective tool for an individualized prediction model. In fact, recent work highlights that machine-learning risk-stratification models have “tremendous utility” for guiding personalized care and decision making, underscoring the potential impact of our findings.

## 4. Discussion

In the study, machine learning algorithms random forests (RF), support vector machines (SVM), multi-layer perceptron (MLP), multi-layer perceptron (MLP_PT, i.e., using the PyTorch library), naive Bayes (NB), and TabPFN have been used to investigate the effect of gallstone risk factors on the presence of any malignancy in those with gallstone disease. The advantage of our study is that it includes a number of data and compares different algorithms with real clinical data using different comparison criteria. No study has been found in this area using these methods.

Our study demonstrates that machine learning (ML) algorithms, particularly TabPFN, are highly effective in predicting malignancy associated with gallstone disease, achieving superior performance across several evaluation metrics such as specificity, accuracy, ROC AUC, Kappa, and Pearson scores. Among the predictive factors identified, the history of HRT use, advanced age (≥55 years), and higher BMI stood out as the most impactful. These findings align with and expand upon the existing literature in several key aspects.

Previous studies have highlighted the role of age and BMI in the risk of malignancy and gallstone formation. For instance, ref. [7] demonstrated that advanced age and obesity were significant predictors of gallstone-related complications, including malignancies, corroborating our findings that age (≥55 years) and BMI are primary risk factors. Similarly, the role of HRT in influencing bile composition and gallstone formation has been documented in studies like that of where postmenopausal women undergoing HRT exhibited a higher incidence of biliary diseases, aligning with our finding that HRT history is a top predictor of malignancy [8].

Our study also highlights the effectiveness of advanced ML algorithms such as TabPFN in predictive analysis. While random forest (RF) and support vector machines (SVMs) have been widely used in medical prediction tasks, our results demonstrate the superior performance of TabPFN, particularly in specificity and accuracy metrics. This is consistent with recent advancements in ML research where ensemble and probabilistic models outperform traditional classifiers in complex biomedical datasets [9].

The impact of hyperparameter tuning in optimizing ML performance, as evidenced by our results, has also been reported in studies such as [10], where individualized tuning significantly improved the performance of ensemble algorithms [10]. Our findings that Kappa and Pearson metrics require fewer ensemble configurations are particularly relevant for practical applications in clinical settings, where computational efficiency is crucial.

From a clinical perspective, identifying risk factors such as age, BMI, and HRT history can assist in the early detection and management of malignancy in patients with gallstone disease. For example, integrating these ML-driven insights into routine clinical workflows could enable targeted screening and personalized management strategies. Furthermore, our findings regarding the predictive capability of TabPFN and RF algorithms suggest that ML could be a valuable tool in clinical decision-making, especially for stratifying high-risk patients.

Another study in the literature stated that machine learning and artificial intelligence methods have been used frequently by researchers recently. In their research, they compared 21 machine learning algorithms using data sets containing six different domains. The performances of the models were evaluated with accuracy, balanced accuracy, F-score, AUC, root mean square error, r squared, and adjusted r-squared [11]. As a result of the analysis, they concluded that the best machine learning method is random forest. In our study, the TabPFN algorithm [2] was used. This algorithm, which is a new method for tabular classification, is different from other machine learning methods. A study comparing the methods we used could not be found in the literature. As a result of our study, it was observed that the machine learning method providing the highest success criterion was TabPFN [2].

A study conducted among American Pima Indians identified a significant association between gallstone disease and elevated overall mortality, particularly cancer-specific mortality, with individuals exhibiting more than a sixfold increased risk of cancer-related death [12]. However, the elevated overall mortality rate could not be fully attributed to cancer-related deaths, indicating that other underlying factors may contribute. While gallstones are recognized as a major risk factor for gallbladder cancer, the incidence of this malignancy remains relatively low [13].

Gallstone disease has also been implicated in the development of malignancies beyond the biliary tract [14,15,16]. Digestive system cancers, which include malignancies of the esophagus, stomach, small intestine, colon, rectum, liver, gallbladder, bile ducts, and pancreas, accounted for an estimated 290,000 new cases and over 140,000 deaths in the United States in 2013 [17]. In developing countries, these cancers rank among the top five most frequently diagnosed malignancies in both men and women. Identifying risk factors for these cancers is critical for designing effective prevention and early detection strategies. Although gallstones have a well-established link to biliary tract cancers [18], their association with digestive system cancers outside the biliary tract remains insufficiently defined.

Gallstones are a common gastrointestinal issue, ranking as the second most frequent discharge diagnosis in U.S. hospitals in 2009, with over 300,000 physician visits attributed to the condition that year [19]. The complications of gallstone disease, such as inflammation in the gallbladder, biliary ducts, liver, and pancreas, are significant [20]. Given the well-established connection between chronic inflammation and cancer [21], gallstones may play a role in increasing the risk of various digestive system malignancies [22]. While cholecystectomy can alleviate inflammation associated with gallstones [23], it may alter bile exposure to other digestive organs, such as the stomach, esophagus, and small intestine, and potentially influence cancer risk through changes in metabolic hormone levels [24].

Epidemiological studies exploring the relationship between gallstone disease and malignancy have yielded mixed findings. A Swedish case-control study reported that cholelithiasis or cholecystectomy was more than twice as common among younger women who died of cancer compared to those who died of other causes, while no such association was observed in men or older women [14]. Similarly, a Finnish study found a higher frequency of prior cholecystectomy among abdominal cancer patients compared to the general population [15]. However, a study conducted in Rochester, MN, reported no significant increase in the incidence of gastrointestinal malignancies, excluding gallbladder cancer, in individuals with gallstones or a history of cholecystectomy compared to the general population [16].

In the present study, we utilized advanced statistical methods to examine factors contributing to the potential association between gallstone disease and malignancy. Our findings revealed a relationship between malignancies and both ultrasound-diagnosed gallstones and prior cholecystectomy. However, this association could not be entirely explained by conventional risk factors, emphasizing the need for further research to elucidate the underlying mechanisms.

As a result, the application of the TabPFN method—proposed in 2022—in this study and the success of this method have been shown in the data, and we think that this study, which is clinically important and has not been found in any research, will be a pioneer for researchers. However, as a further step of this study, simulation studies and methods can be compared for data sets with different data properties.

In summary, our findings underscore the potential of ML algorithms, particularly TabPFN, in predicting malignancy risk associated with gallstone disease. By identifying critical risk factors such as HRT history, age, and BMI, our study contributes to a growing body of evidence that leverages data-driven approaches for improved clinical outcomes.

## 5. Conclusions

Prediction model in clinical practice: The models identify a high-risk gallstone patient profile (female, ≥55 years old, high BMI, on HRT). Clinicians should consider more proactive monitoring for such patients. For example, periodic abdominal ultrasound or liver function tests could be performed in older, obese women with gallstones and history of HRT to detect early malignancies. In select high-risk cases, earlier referrals for cholecystectomy or imaging may be justified to rule out occult neoplasia.

Future model development and validation: Further research should focus on external validation in diverse populations (multi-center cohorts, different ethnic groups) to ensure generalizability. Incorporating additional variables (genetic markers, laboratory values, imaging features) and testing alternative algorithms (other tabular deep-learning or ensemble methods) may improve accuracy.

Simulation and prospective studies: Implement simulation analyses to evaluate model performance under varying prevalence of malignancy and missing data scenarios. Ultimately, a prospective study or clinical trial could test whether using the model to guide surveillance reduces cancer morbidity or mortality.

Broader applications: Given the strong performance of TabPFN on moderate-sized clinical data, this approach could be extended to related questions, such as predicting other gallstone-related complications, thereby contributing to precision medicine in hepatobiliary care.

## Figures and Tables

**Table 1:** Descriptive statistics of variables.

		Frequency	Percent
Any Malignancy	Yes	781	78.2
	No	218	21.8
Gender	Male	213	21.3
	Female	786	78.7
BMI	<30	415	41.5
	≥30	584	58.5
CROHN	Yes	996	99.7
	No	3	0.3
DM	Yes	465	46.5
	No	534	53.5
Liver Cirrhosis	Yes	940	94.1
	No	59	5.9
Hashimoto	Yes	886	88.7
	No	113	11.3
HCV	Yes	927	92.8
	No	72	7.2
HRT Use	Yes	948	94.9
	No	51	5.1
Oral Contraceptive Use	Yes	958	95.9
	No	41	4.1
Ceftriaxone Use	Yes	755	75.6
	No	244	24.4
Statin Use	Yes	545	54.6
	No	454	45.4
Cholecystectomy	Yes	521	52.2
	No	478	47.8
Graves	Yes	941	94.2
	No	58	5.8

**Table 2:** The best scores of each ML algorithm. Bold entries represent the best score for each metric where underlined entries represent the second-best scores.

Alg.\Score	Sensitivity	Specificity	Accuracy	AUC	Kappa	Pearson
RF	0.626	*0.826*	*0.782*	0.716	0.413	0.428
SVM	0.555	0.597	0.588	0.576	0.127	0.333
MLP	0.72	0.703	0.707	0.695	0.336	0.354
MLP_PT	0.254	0.823	0.696	0.554	0.109	0.093
NB	**0.747**	0.713	0.72	0.729	0.361	0.391
TabPFN	0.691	**0.847**	**0.814**	**0.768**	**0.495**	**0.503**

**Table 3:** Hyperparameters of the random forest model that maximize each score.

	Hyperparameters	Sensitivity	Specificity	Accuracy	Kappa	Pearson
Random Forest Model	Max Dept	48	33	40	17	42
	# of Bins	13	15	8	13	11
	# of Estimators	9	41	32	35	27
Support Vector Machine Model	C	1326.587	7.41 × 10^−6^	1785.877	2991.139	1775.587
Multi-Layer Perceptron Model	Learning Rate	0.405	0.005	0.004	0.052	0.15
	Size of 1st Hidden Layer	78	69	82	76	58
	Size of 2nd Hidden Layer	63	79	35	90	42
	Batch Size	2	8	20	2	17
Multi-Layer Perceptron (via PyTorch) Model	Learning Rate	0.441	0.004	0.225	0.188	0.129
	Size of 1st Hidden Layer	89	67	55	75	87
	Size of 2nd Hidden Layer	64	52	92	33	80
	Batch Size	1	26	90	44	1
Naive Bayes Model	Smoothing parameter	0.0012	0.0001	0.182	0.5802	0.4193
TabPFN	# of Ensemble Configurations	37	50	37	8	6

## Data Availability

The original contributions presented in this study are included in the article. Further inquiries can be directed to the corresponding author(s).
